# A Context-Based Analgesia Model in Rats: Involvement of Prefrontal Cortex

**DOI:** 10.1007/s12264-018-0279-6

**Published:** 2018-09-03

**Authors:** Lingchi Xu, Yalan Wan, Longyu Ma, Jie Zheng, Bingxuan Han, Feng-Yu Liu, Ming Yi, You Wan

**Affiliations:** 1 Neuroscience Research Institute, Peking University, Beijing, 100083 China; 2 Department of Clinical Medicine, Zhongshan School of Medicine, Sun Yat-Sen University, Guangzhou, 510080 China; 3 Department of Neurobiology, School of Basic Medical Sciences, Peking University, Beijing, 100083 China; 4 Key Laboratory for Neuroscience, Ministry of Education/National Health and Family Planning Commission, Peking University, Beijing, 100083 China; 5 Co-innovation Center of Neuroregeneration, Nantong University, Nantong, 226001 China

**Keywords:** Context-based analgesia, Placebo analgesia, Pain, Hot-plate test, Cognition modulation, Opioid system, Prefrontal cortex

## Abstract

Cognition and pain share common neural substrates and interact reciprocally: chronic pain compromises cognitive performance, whereas cognitive processes modulate pain perception. In the present study, we established a non-drug-dependent rat model of context-based analgesia, where two different contexts (dark and bright) were matched with a high (52°C) or low (48°C) temperature in the hot-plate test during training. Before and after training, we set the temperature to the high level in both contexts. Rats showed longer paw licking latencies in trials with the context originally matched to a low temperature than those to a high temperature, indicating successful establishment of a context-based analgesic effect in rats. This effect was blocked by intraperitoneal injection of naloxone (an opioid receptor antagonist) before the probe. The context-based analgesic effect also disappeared after optogenetic activation or inhibition of the bilateral infralimbic or prelimbic sub-region of the prefrontal cortex. In brief, we established a context-based, non-drug dependent, placebo-like analgesia model in the rat. This model provides a new and useful tool for investigating the cognitive modulation of pain.

## Introduction

Pain and cognition inherently influence each other: pain can negatively affect cognitive performance, whereas cognitive modulation occurs in painful situations, for example as placebo and nocebo effects [1–3]. Recent studies have shown that cognitive functioning predicts the occurrence of post-surgical pain [4]. Some therapeutic interventions for pain, such as cognitive behavioral therapy, psychological consultation, and meditation, also target the cognitive-evaluative dimension of pain. However, the mechanisms underlying these interactions are not fully understood.

Efforts have been made to build rodent models of the cognitive modulation of pain, as well as cognition-mediated placebo analgesia [5–8], but these different training protocols are all based on analgesics that induce physiological changes and stress reactions. Based on the previous descriptions, in the present study we set out to build a rat model of context-based analgesia by matching two different contexts to a high or low testing temperature in the hot-plate test. Then the involvement of the opioid system in the context-based analgesia was identified by naloxone injection.

Previous studies have shown that the activation of prefrontal glutamatergic neurons enhances recognition memory [9]. In addition, the activation of archaerhodopsin (Arch, a light-activated inhibitory proton pump) or channel rhodopsin 2 (ChR2, a light-activated excitatory cation channel) in parvalbumin (PV)-positive interneurons in the prefrontal cortex (PFC) decreases or increases pain responses, respectively [10]. Based on this, we hypothesized that the effects of cognition-mediated analgesia could be influenced by activation or inhibition of the PFC.

## Materials and Methods

### Animals

Adult female Sprague-Dawley rats weighing 250–300 g at the beginning of the experiment were provided by the Department of Experimental Animal Sciences, Peking University Health Science Center. Male rats were excluded because of the risk of the testicles being burned in the hotplate test. The rats were housed 4–6 per cage in a temperature- and light-controlled room under a 12:12 h light:dark cycle with water and food provided *ad libitum*. The animals were handled and habituated for 3–5 days before experiments. All experimental procedures were conducted in accordance with the guidelines of the International Association for the Study of Pain, complied with the ARRIVE guidelines [11], and were approved by the Animal Care and Use Committee of our University.

### Hot-Plate Test

The hot-plate apparatus with a 30 × 30 × 30 cm^3^ Plexiglas chamber was located in a quiet room. The actual temperature of each test trial was as indicated below. The cut-off time was 30 s for the low temperature trials (LT, 48 ± 0.5°C), and 30 s for the high temperature trials (HT, 52 ± 0.5°C) to avoid possible plantar injury. The inter-trial interval was at least 10 min.

### Establishment of a Rat Model of Context-Based Analgesia

**Fig. 1:**
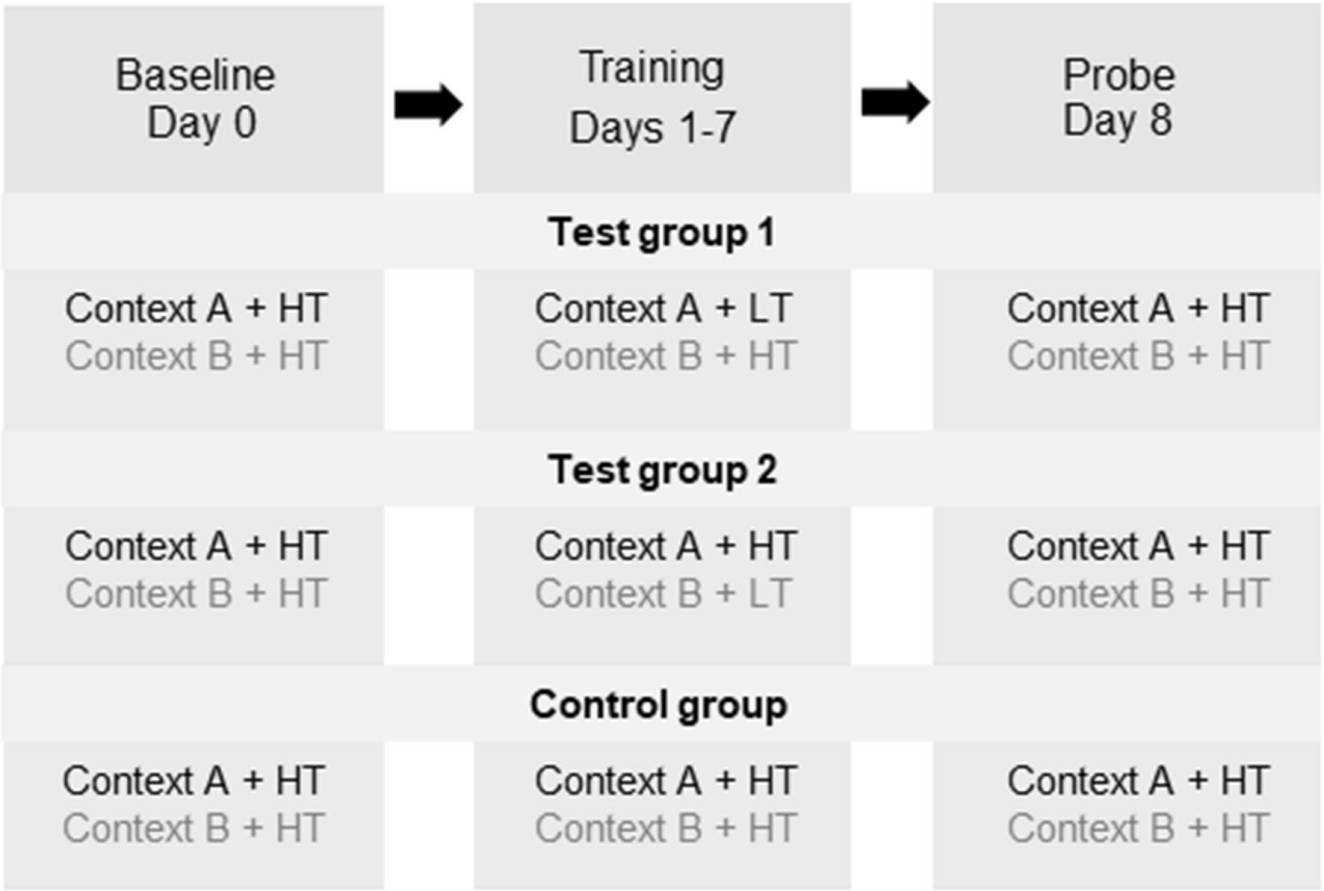
Training and probe flowchart for the context-based analgesic effect in rats. Three groups were trained and tested in the three-phase experimental paradigm (Baseline: left column; Training: middle column; Probe: right column). Contexts A (black) and B (grey) were in the same room but brightly- and dimly-lit, respectively. HT, high temperature (52°C); LT, low temperature (48°C).

The training and testing procedures are shown in Fig. 1. The hot-plate test was performed in two different contexts in the same room: Context A was brightly lit (500–600 lumen) whereas Context B was dimly lit (1–2 lumen). After habituation to the apparatus for 2 days, baseline testing at the HT was performed on day 0. Paw licking latencies (PLLs) were averaged from 3 trials in each context. The trial sequence was pseudo-randomized so that no more than two consecutive trials were performed in the same context. The training phase was during days 1–7. Three groups (Test groups 1 and 2, and the Control group) were used to investigate the potential influences of different context and temperature combinations. These three groups were performed separately with three batches of animals. In Test group 1, Context A was matched with the LT and Context B with the HT. In Test group 2, Context A was matched with the HT and Context B with the LT. In the Control group, both contexts were matched with the HT. To strengthen the influence of contextual cues, the rat was placed in the corresponding context for 5 min before each trial as pre-exposure. On day 8, a probe test was performed as on day 0.

Training and testing (baseline and probes) were run by different experimenters to ensure testing blinded from groupings. This blinded approach also applied to all the subsequent experiments.

### Naloxone Injection in the Hot-Plate Test

To determine whether opioids are involved in context-based analgesia, manipulations on days 0–8 were identical to those described above. Context A was matched with the LT and Context B with the HT. On day 9, animals were randomly divided into two groups. Half of the animals received an intraperitoneal (i.p.) injection of naloxone (an opioid antagonist) dissolved in normal saline (NS) at 5 mg/kg body weight while the other half were injected with an equal volume of NS. Thirty minutes after injection, all rats received the probe test again as on day 8. This was repeated on day 10 with each animal receiving an injection of the other solution (NS or naloxone). Data from days 9 and 10 were pooled as probe 2 for evaluating the effects of naloxone antagonism. The behavioral experimenters were blind to the solutions injected.

### Surgical Procedure

Female Sprague-Dawley rats weighing 250–300 g were used in the optogenetic experiments. Each rat was anesthetized with 0.5% pentobarbital sodium (100 mg/kg, i.p.; Merck, Darmstadt, Germany) and placed in a stereotaxic frame. pAAV-CaMKIIa-hChR2-EYFP (2.7 × 10^13^ viral genomes (vg)/mL) or pAAV-CaMKIIa-ArchT-EYFP (2.7 × 10^13^ vg/mL) (Heyuan, Shanghai, China) was injected bilaterally into the prelimbic (PL; left side, AP +3.0, ML −1.8, DV −4.0 mm, tilted 20°; right side, AP +3.0, ML −0.5, DV −3.5 mm, vertical) or infralimbic cortex (IL; left side, AP +3.0, ML −1.8, DV −5.1 mm, tilted 20°; right side, AP +3.0, ML −0.5, DV −4.6 mm, vertical) through two small burr holes in the skull, where optical fibers (NA = 0.37, Φ = 200 mm; Fiblaser, Shanghai, China) were subsequently implanted. Expression of ChR2 and ArchT driven by the cell-type-specific promoter CaMKII was then observed in the glutamatergic PFC neurons. The optical fibers were fixed to the skull with dental cement (New Century, Shanghai, China).

### Optogenetic Stimulation During the Hot-Plate Test

The context-related behavioral training tests were performed 30 days later to allow for viral expression. The training paradigms were identical to those described above. Context A was matched with the LT and Context B with the HT. On day 9, animals were randomly divided into two groups of equal numbers. Light was delivered and the animal was put into the hot plate in one group, while the other group without light delivery in tests. A laser generator was connected to the bilateral optic fibers for at least 5 min before light delivery. The LED was turned off at the moment the rat licked its paw. This test was repeated on day 10 with exchange of the optogenetic stimulation in rats for self-contrast. hChR2- and ArchT-expressing neurons were stimulated with blue (25-ms pulses, 20 Hz, 6–9 mW, 473 nm) and yellow (25-ms pulses, 20 Hz, 6–9 mW, 589 nm) light during the hot-plate tests, respectively. The power output delivered was confirmed as 6–9 mW for each stimulation session using a power meter (Thorlabs, Newton, NJ).

### Brain Slice Preparation for Whole-Cell Patch-Clamp Recording

Female Sprague-Dawley rats (250–300 g) were used for whole-cell patch clamp recording. The rats used for patch clamp were not the same as those used for the context-based analgesia experiments (*n* = 5 for the optogenetic-activation group; *n* = 6 for the optogenetic-inhibition group). Each rat was anesthetized with pentobarbital sodium (40 mg/kg, i.p.). The brain was removed within 1 min and submerged in ice-cold artificial cerebrospinal fluid (aCSF) containing (in mmol/L): 125.0 NaCl, 2.5 NaH_2_PO_4_, 2.6 KCl, 1.3 CaCl_2_, 21.0 NaHCO_3_, 0.9 MgCl_2_, and 3.5 glucose. Coronal slices (thickness, 400 μm) that contained the PL/IL cortices were then cut on a Vibroslice (1,000+, Pelco 102; Ted Pella Inc., Redding, CA). The anatomical locations of the PL/IL cortices were confirmed on the basis of a rat brain atlas (Paxinos and Watson, 2007) and have been previously reported by our laboratory [12]. Before a single slice was transferred to a submerged recording chamber, slices were incubated in an oxygenated aCSF bath at room temperature for at least 1 h. The chamber was perfused with aCSF (2–3 mL/min) using a pump (Peri-Star 291, World Precision Instruments, Sarasota, FL). All experiments were performed at room temperature [13].

### Whole-Cell Patch Clamp Recordings

Each slice was viewed under a microscope (Axioskop Fsmot; Zeiss, Jena, Germany) equipped with infrared differential interference contrast optics. Pyramidal cells in the bilateral PL/IL cortices were identified through a 40× water-immersion lens.

Voltage and current signals were recorded from EYFP-expressing pyramidal cells using an Axon 200B amplifier (Axon Instruments, Union City, CA). Action potentials (APs) and inhibitory post-synaptic potentials (IPSPs) were recorded in the current-clamp mode. The holding voltage for the excitatory post-synaptic currents (EPSCs) and APs was the same (–70 mV), and 0 mV for recording IPSCs. The aCSF contained the following (in mmol/L): 124 NaCl, 26 NaHCO_3_, 3.0 KCl, 1.0 NaH_2_PO_4_, 1.3 MgCl_2_, 1.5 CaCl_2_, 20 *D*-glucose, saturated with 95% O_2_ and 5% CO_2_. The pipette solution contained (in mmol/L) 120 potassium gluconate, 10 KCl, 4 ATP-Mg, 0.3 GTP, 10 HEPES, and 0.5 EGTA (pH 7.2, 270–280 mOsm with sucrose).

### Histology

The procedure was the same as in our previous report [14]. After all behavioral tests, each rat was deeply anesthetized and perfused with 4% paraformaldehyde in phosphate buffer. IL/PL sections (20 μm thick) were cut coronally on a freezing microtome and used to identify the expression of EYFP and the locations of the optical fibers. Data from animals with incorrect location were excluded from further statistical analysis.

### Statistics

**Fig. 2:**
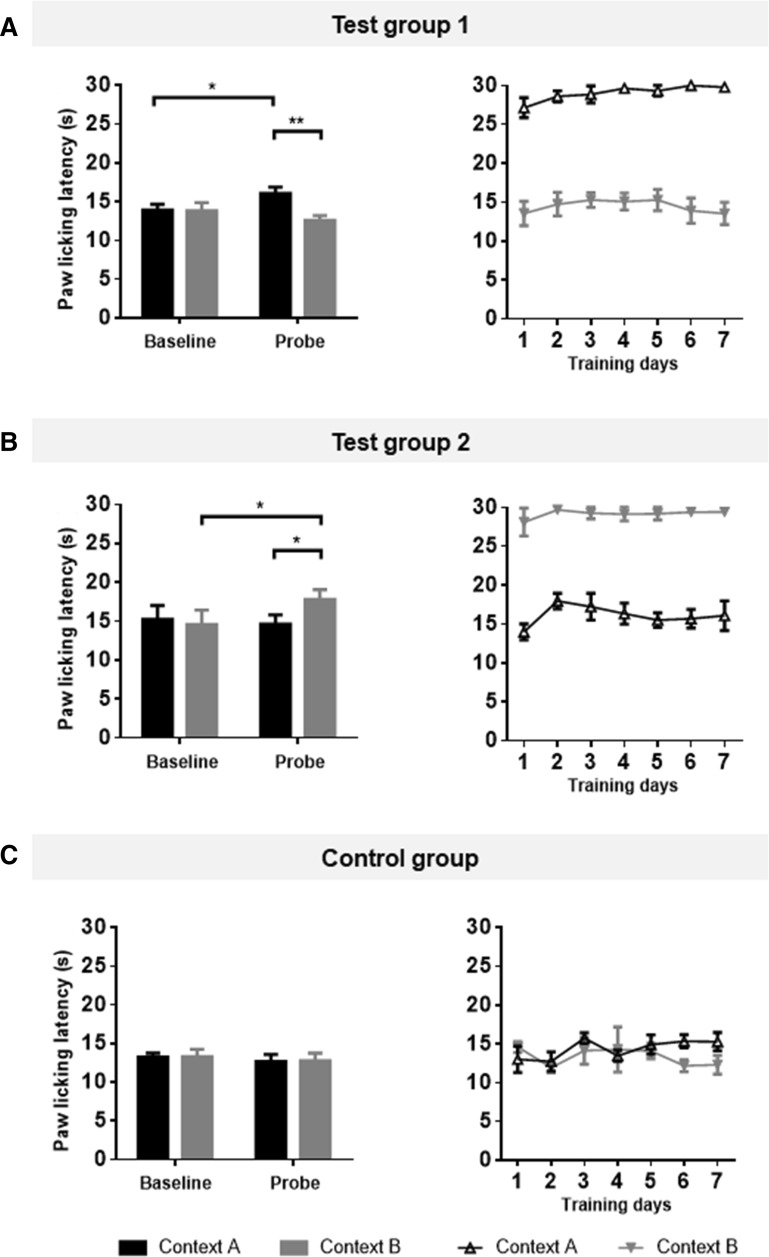
Establishment of the context-based analgesia model in rats. Three groups were trained and tested in the three-phase experimental paradigm. Context A (black) was a bright room whereas Context B (grey) was a dim room. Paw licking latencies (PLLs) of Test group 1 (**A**), Test group 2 (**B**), and Control group (**C**) in the baseline, training and probe phases are shown. The different contexts modulated pain perception with the same hot-plate temperature in the probe test. The context-based analgesic effect was indicated by the differential PLLs between baseline and the probe test with the same context and hot-plate temperature. *n* = 8, *P < 0.05, **P < 0.01, paired *t* test.

All data are presented as the mean ± SEM. Analysis of context-based analgesia and the effects of naloxone on the hot-plate test was performed with paired Student’s *t* test after a Gaussian distribution was found, except for the control group in Fig. 2, where the Wilcoxon matched pairs test was used. *P* < 0.05 was considered to be statistically significant.

## Results

### Establishment of Context-Based Analgesia Rat Model

Baseline testing (day 0) at the HT revealed no differences in PLL between contexts in all three groups (Test group 1: *t* = 0.09, *n* = 8; Test group 2: *t* = 0.36, *n* = 8; Control group: *W* = 3; all *P* > 0.05) (Fig. 2).

During the 7 days of training, PLLs in the HT-matched context (Context B for Test group 1 and Context A for Test group 2) were shorter than those in the LT-matched context (Context A for Test group 1 and Context B for Test group 2, Fig. 2A, B). In the Control group in which the same temperature was used, PLLs were comparable between contexts (Fig. 2C).

In the probe trial on day 8, the PLLs of rats that stayed in the context originally matched with the HT (Context B for Test group 1 and Context A for Test group 2) were significantly shorter than those with the LT (Context A for Test group 1 and Context B for Test group 2) (Test group 1: *t* = 4.61, *P * < 0.01; Test group 2: *t* = 2.94, *P  *< 0.05) (Fig. 2A, B). In contrast, the PLLs in the control group did not significantly differ between contexts (*W* = 3, *P* > 0.05) (Fig. 2C).

The PLLs of probe tests in the context matched with the LT (Context A for Test group 1 and Context B for Test group 2) were significantly longer than baseline (Test group 1: *t* = 2.65; Test group 2: *t* = 2.94, both *P* < 0.05) (Fig. 2A, B), while the PLLs of probe tests in the context originally matched with the HT did not differ from baseline (Test group 1: *t* = 1.24; Test group 2: *t* = 0.29, both *P* > 0.05) (Fig. 2A, B). These results indicate a clear cognition-mediated analgesic effect in the Test groups, induced by behavioral training with two contexts. In addition, different combinations of contexts and temperatures had no significant effect on the results.

### Naloxone Confirmation of Context-Based Analgesia

It is well known that classical cognition-pain modulation is very similar to placebo analgesia, which is endogenously opioid-dependent [6, 15]. So we next performed naloxone blockade experiments to determine whether the above context-based analgesia is dependent on cognitive modulation.

**Fig. 3:**
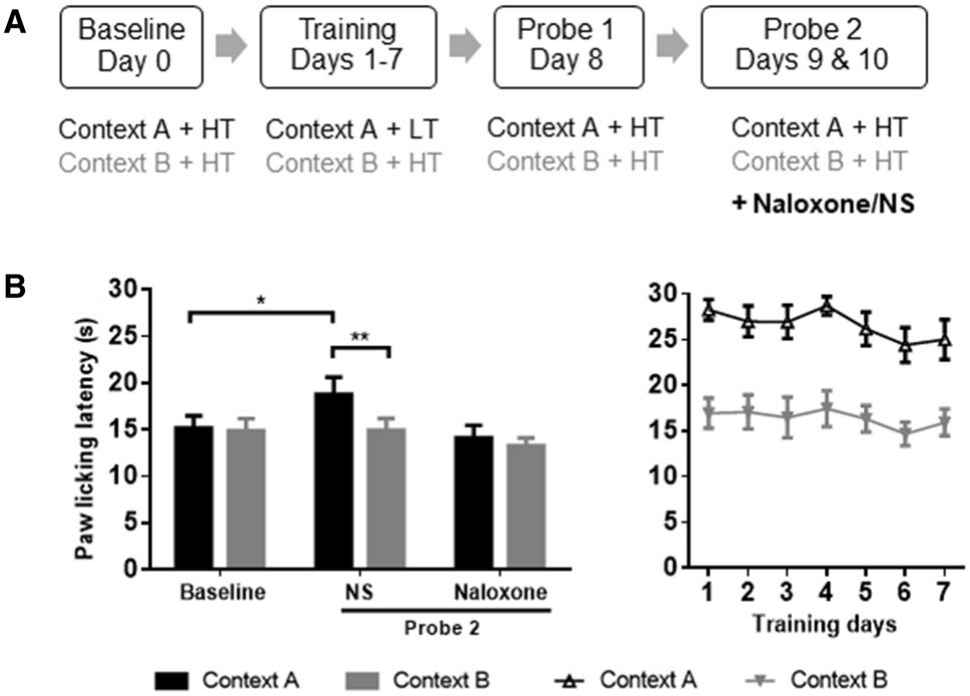
Naloxone blockade of the context-based analgesic effect in rats. **A** Training and testing paradigm. **B** Naloxone injection affected PLLs in the hot-plate test. In contrast, the context-based analgesia was not influenced by saline injection. Context A, black; Context B, grey; HT, high temperature; LT, low temperature. *n* = 10, *P < 0.05, **P < 0.01, paired *t* test.

Behavioral training yielded a context-based analgesic effect similar to that described above (Fig. 3). Probe test on day 8 indicated a clear and stable difference in context-dependent pain perception between contexts (*t* = 2.58, *P* < 0.05, *n* = 10). The PLLs of probe test 1 in context A (matched with LT in training) were significantly longer than baseline (*t* = 3.06, *P* < 0.05), which demonstrated a correlation between the context matched with the LT and context-based analgesia, while the PLLs in context B (matched with the HT in training) did not differ significantly between probe test 1 and baseline (*t* = 0.69, *P* > 0.05).

Interestingly, injection of naloxone abolished this context-based analgesic effect (*n* = 10, NS: *t* = 3.86, *P* < 0.01; naloxone: *t* = 0.90, *P* > 0.05) (Fig. 3B), suggesting that the opioid system is involved in mediating this cognitive modulation of analgesia. Meanwhile, the PLLs (context A) in the saline group were longer than baseline (*t* = 2.50, *P * < 0.05) (Fig. 3B). These results indicate that the context-induced analgesia effect depends on the endogenous opioid system.

### Effective Activation/Inhibition of Pyramidal Neurons in PL/IL Cortices

**Fig. 4:**
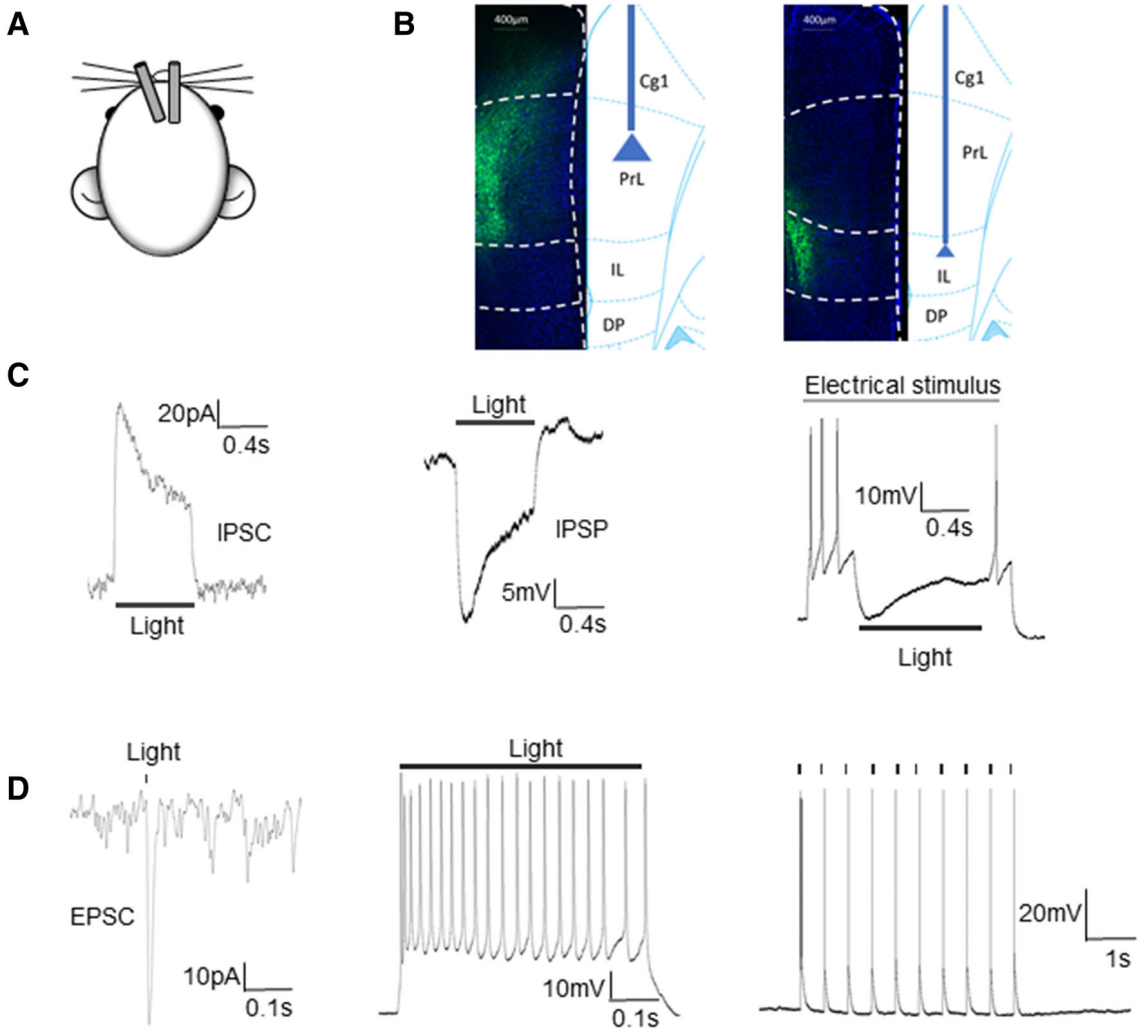
Confirmation of optogenetic inhibition or inhibition of neuronal firing in pyramidal neurons. **A** Schematic of the implanted optic fibers: in the left hemisphere tilted 20°, and vertical on the right side. **B** EYFP expression in excitatory PL/IL neurons after viral injection. **C** Examples of yellow light-induced outward current and membrane hyperpolarization in a neuron expressing ArchT. An IPSC (left), IPSP (middle), and inhibition of APs were induced by the yellow light stimulation. **D** Example of a blue light-evoked EPSC recorded in an EYFP-tagged ChR2-expressing neuron (left). Current clamp recordings under either continuous blue-light stimulation or in response to blue light delivered at interpulse intervals of 0.5 s. The pulse-locked neuronal firing was induced by the blue light, confirming the expression and function of ChR2 in the pyramidal neuron (middle and left).

Optogenetic manipulation with hChR2 and Arch has been widely used to activate or inhibit specific types of neurons. The hChR2 or Arch gene can be selectively expressed in specific neurons with a neuronal type-specific promoter [10, 13, 14, 16]. We also used fluorescent staining of pyramidal neurons to confirm the localization and expression of pAAV-CaMKIIa-hChR2-EYFP and pAAV-CaMKIIa-ArchT-EYFP in the bilateral PFC subregions PL and IL (Fig. 4B), as in our previous report [14].

In this study, whole-cell patch clamp recordings were performed to determine whether hChR2 and ArchT were expressed in glutamatergic neurons with the CaMKIIa promoter. The recordings from ArchT-expressing pyramidal neurons revealed that yellow-light (589 nm) stimulation not only evoked IPSCs and IPSPs, but also inhibited AP firing during current injection through the micropipette (Fig. 4C). hChR2-expressing glutamatergic neuronal activity was recorded in brain slices. Blue-light (473 nm) stimulation induced strictly pulse-locked APs in neurons (Fig. 4D). Thus, we confirmed the expression and function of hChR2 and ArchT in pyramidal neurons under the control of the CaMKIIa promotor.

### Optogenetic Activation of the PL or IL Cortex Eliminates the Context-Based Analgesia

**Fig. 5:**
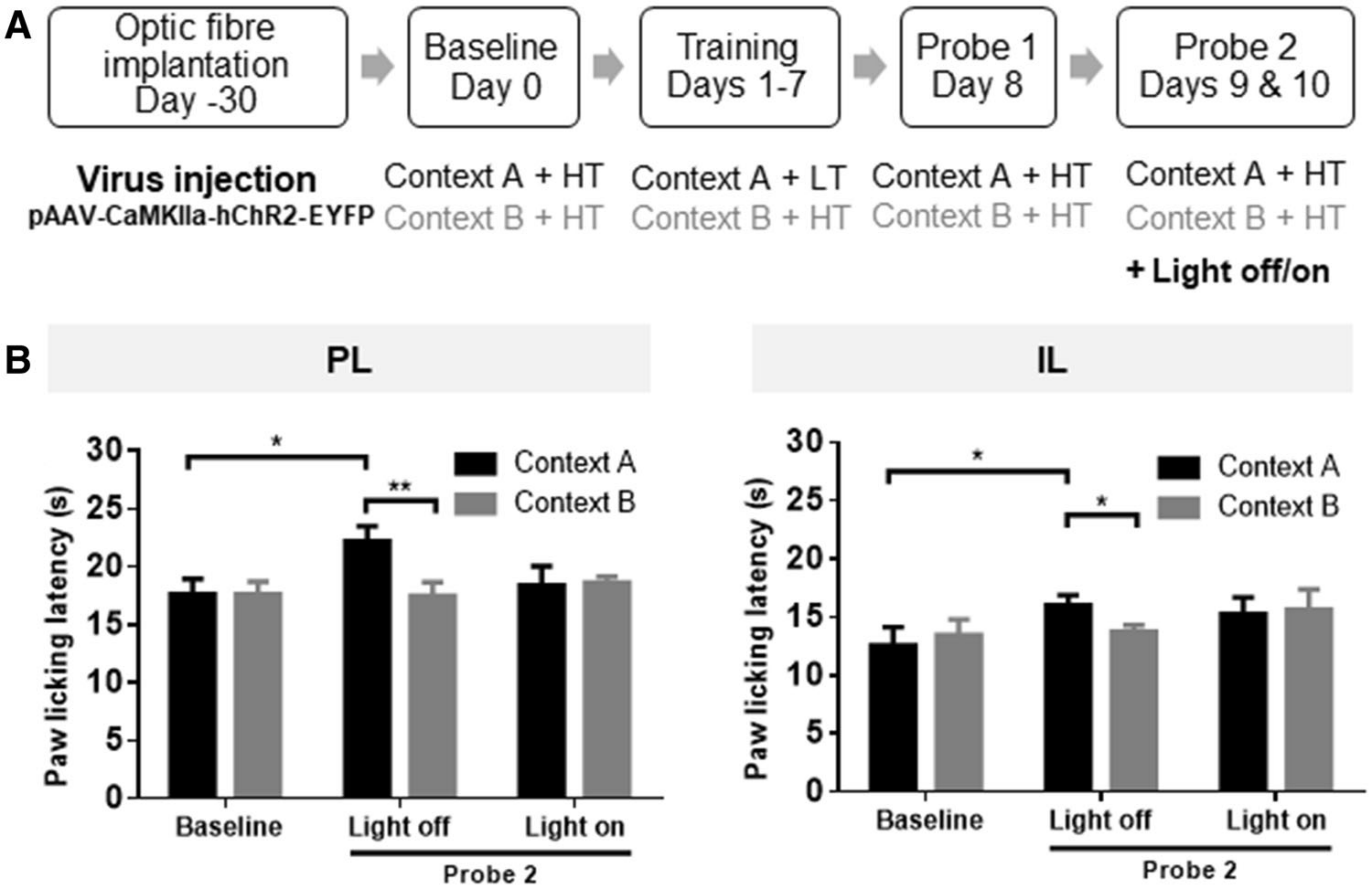
Optogenetic activation of either PL or IL excitatory neurons blocked the context-based analgesic effect in rats. **A** Training and probe paradigm. **B** Optogenetic activation of neurons in either PL or IL cortex affected PLLs in the hot-plate test. Note that the context-based analgesia was significantly decreased with LED-on but not with LED-off. Context A, black; Context B, grey; HT, high temperature; LT, low temperature. *n* = 10 in both PL and IL groups, *P < 0.05, **P < 0.01, paired *t* test.

To determine whether the bilateral PL or IL cortex plays a role in context-based analgesia in rats, we used an optogenetic technique that enables specific activation of glutamatergic neurons. The behavioral training paradigm is shown in Fig. 5A.

Probe test 1 indicated a clear and stable context-dependent difference in pain perception between contexts in the PL group (*t* = 3.39, *P * < 0.01, *n* = 10). The PLLs of probe test 1 in context A were significantly longer than baseline (matched with LT in training) (*t* = 2.49, *P * < 0.05). These results indicated that an analgesic effect based on cognition of different contexts was successfully established in rats.

Optogenetic activation of pyramidal cells in the PL abolished this context-based analgesic effect (*n* = 10, LED-off: *t* = 4.22, *P* < 0.01; LED-on: *t* = 0.12, *P* > 0.05) (Fig. 5B), suggesting that the PL cortex is responsible for mediating the context-based analgesic effect. A significant increase of PLLs (context A) in the LED-off group was found compared with baseline (*t* = 2.30, *P* < 0.05) (Fig. 5B).

Similarly, optogenetic activation of pyramidal cells in the IL cortex (LED-on) also abolished this context-based analgesic effect (*n* = 11, LED-off: *t* = 2.57, *P* < 0.05; LED-on: *t* = 0.29, *P* > 0.05) (Fig. 5B).

Together, these data indicate that pyramidal neurons in the prefrontal cortex (in the PL and IL cortices) participate in non-drug dependent, context-based analgesia and that optogenetic activation of these neurons eliminates the established context-based analgesic effect in rats.

### Optogenetic Inhibition of the PL or IL Cortex also Eliminated the Context-Based Analgesia

**Fig. 6:**
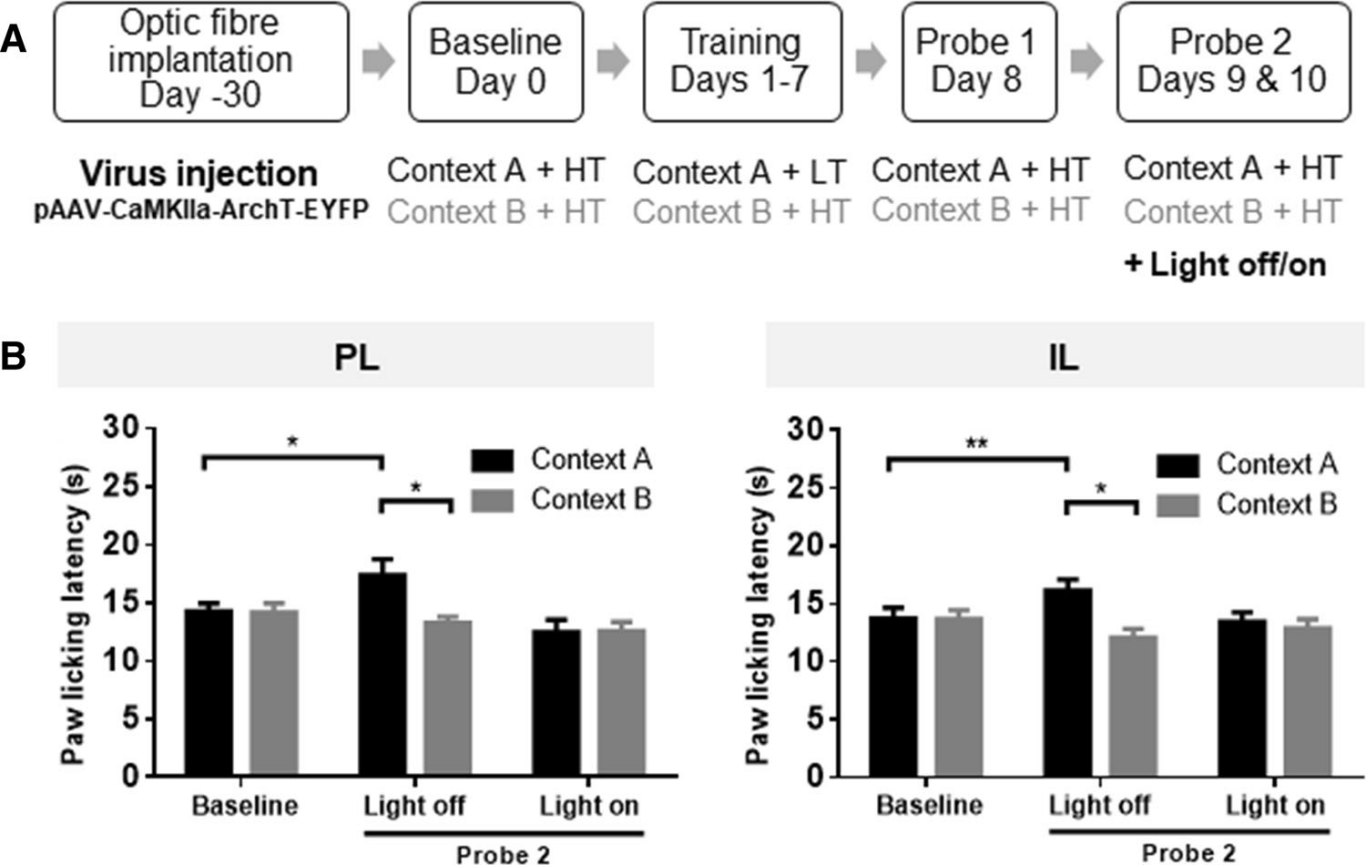
Optogenetic inhibition of either PL or IL excitatory neurons blocked the context-based analgesic effect in rats. **A** Training and probe paradigm. **B** Optogenetic inhibition of neurons in either the PL or IL cortex abolished the PLL difference between the two contexts in rats showing context-based analgesia. Note that PLLs were not influenced at light-off during the hot-plate test. Context A, black; Context B, grey; HT, high temperature; LT, low temperature. *n* = 10 in the PL group and *n* = 11 in the IL group, *P  < 0.05, paired *t* test.

The behavioral training paradigm is shown in Fig. 6A. Optogenetic inhibition of pyramidal neurons in the PL cortex blocked the context-based analgesic effect (*n* = 10, LED-off: *t* = 2.58, *P* < 0.05; LED-on: *t* = 0.12, *P* > 0.05) (Fig. 6B), suggesting that the PL cortex is responsible for mediating this effect. A significant increase of PLLs (context A) in the LED-off group occurred compared with baseline (*t* = 2.46, *P* < 0.05) (Fig. 6B).

Similar to the PL cortex, optogenetic inhibition of pyramidal neurons in the IL cortex also blocked the context-based analgesic effect (*n* = 11, LED-off: *t* = 2.90, *P * < 0.01; LED-on: *t* = 0.63, *P* > 0.05) (Fig. 6B).

Altogether, these results suggest that pyramidal neurons in the PL and IL cortices participate in the non-drug-dependent, context-based analgesia and that optogenetic inhibition of these neurons eliminates the established context-based analgesia effect in rats.

## Discussion

Pain and cognition have close interactions. In the present study, two different contexts (dark and bright) were matched with high (52°C) or low (48°C) temperature in the hot-plate test during training. Rats showed longer PLLs in trials with the context originally matched to the LT than those to the HT (Fig. 2), indicating successful establishment of a context-based analgesic effect. This model is a type of placebo-like cognition modulation. Naloxone blockade (Fig. 3) showed that this context-based analgesia is a placebo-like phenomenon.

### Animal Model for Cognitive Modulation of Pain

Pain is a highly subjective perceptual experience that can be affected by cognitive processes [17]. Typical examples include the contextual and emotional modulation of pain as well as placebo effects. Neuroimaging studies have revealed several cortical and subcortical substrates of these effects [18–25]. Support for causal relationships between brain activity and pain modulation has been obtained from brain stimulation studies, including transcranial direct current stimulation and transcranial magnetic stimulation [26, 27]. However, more in-depth investigation of the cognitive modulation of pain at the neuronal and molecular levels requires back-translation of human studies to animal models, which is not easily established [7].

Several rodent models have been reported, with different training protocols, analgesics, and methods of pain evaluation [7, 8, 28]. Most of them primarily applied a conditioning strategy, by associating the injection of analgesics with specific visual or contextual cues. Later exposure to these cues yielded conditioned placebo or nocebo effects and/or expectation. Another strategy, developed by Ford *et al.* [29, 30], used novel contexts or objects in the testing chamber to distract the animal’s attention from pain. This model showed attenuated nociceptive behaviors in the second phase of the formalin test.

In the present study, we developed a novel strategy, by matching two different contextual cues to different testing temperatures in the hot-plate test (Fig. 2). The absence of analgesics in the whole procedure mimics many clinical situations where pure cognitive processes, without any medication, are sufficient to modulate pain. In addition, the hot-plate test is a physiological pain test without persistent injury, enabling multiple probing trials in the same subject. This model also allows flexible revision for experimental necessity. For example, analgesics can be incorporated into the training protocol, and the training phase can be prolonged or repeated to consolidate the strength of modulation. Finally, the self-control strategy for evaluating cognitive modulation bypasses daily fluctuations in the pain threshold without affecting cross-subject differences in the modulatory effect.

### Role of the PFC in Context-Based Analgesia

Pain is a distressing experience associated with actual or potential tissue damage with sensory, emotional, cognitive, and social components [31]. The PFC is a crucial integration center for both sensory and emotional pain perception [10, 32–34]. Non-invasive low-frequency repetitive transcranial magnetic stimulation can completely block a context-based analgesic effect [27]. Furthermore, the PFC participates in expectancy-induced changes in subjective pain ratings [35].

In this study, optogenetic light-activation of inhibitory ArchT or excitatory hChR2 in glutaminergic neurons in both the PL and IL cortices blocked the context-related (Figs 5, 6), non-drug-dependent analgesic effect in rats. These results are consistent with a previous report [36]. Interestingly, however, unlike mechanical pain sensitivity, thermal pain sensitivity is changed less by cortical modulation. For example, in a rat model of empathy for pain, which is also dependent on the PL/IL cortices, only mechanical pain hypersensitivity can be demonstrated while thermal pain sensitivity remains unchanged [37, 38]. Intra-PL/IL administration of ethanol has also been demonstrated to result in mechanical pain hypersensitivity but with unchanged thermal pain sensitivity [39].

The differences between the findings of the new model and previous findings may be because the context-based experimental strategy is more dependent on the training-induced experience rather than emotional impact compared with empathy. The mechanisms of expectation-induced analgesia and empathy-induced pain sensitivity may be different. Several studies have shown that activity due to expectation-induced analgesia in the frontal cortex is associated with pain processing and pain modulation [24]. Since ventromedial PFC (including both IL and PL cortices) activity is associated with reward expectation [40], one possible interpretation of our results is that the cognitive function of the PFC plays a more important role in the expectation-related modulation of pain than in pain sensation. Therefore, it is important to consider the possibility that the integrative function of the PFC is decisive in context-based analgesia—either activating or silencing the PFC could influence the PFC homeostasis and thus interfere with the cognitive processing of the contextual modulation of pain. So, the cognitive function of the PFC plays a more important role in pain modulation than in pain sensation.

This study is the first to demonstrate context-based analgesia with pinpoint accuracy to the PL and IL cortices. However, the literature suggests that the left and right PFCs may respond to different executive functions [41]. Thus, further research is needed to investigate the role of the PL and IL cortices in the context-based analgesic effect. Understanding the cognitive modulation of pain like that in context-based analgesia is complex, because it involves multiple brain regions that project to the PFC. Our data demonstrate that the role of the PFC is like an aggregator, thus feasible upstream and downstream structures should also be considered in further studies.

### A Novel Context-Based Analgesia Model Mediated by Expectation in Rats

Both the placebo effect and context-based analgesia are based on previous experience, so we speculate that common features may exist between this new model of context-based analgesia and placebo analgesia. The most well-known theories pertaining to the contextual-modulation analgesic effect are classical conditioning and expectation [42, 43].

Distinct pharmacological mechanisms underlying expectation and conditioning have been revealed by a carefully designed human study, which has shown that expectation triggers endogenous opioids, while conditioning activates specific subsystems not necessarily dependent on the opioid system [44]. Expectation-dependent placebo analgesia mediated by the opioid system has been reliably reported in the literature [45]. Morphine (an opiate analgesic)-induced placebo analgesia is dependent on expectation and can be blocked by opioid antagonists. However, conditioning with nonsteroidal anti-inflammatory drugs elicits opioid-independent analgesia that is only partially mediated by expectation [6, 46].

According to the literature, naloxone is an opioid antagonist used in rodent experiments at a dose of 5 mg/kg. Therefore, 5 mg/kg was selected as an appropriate dose for the current procedure. In our novel model, naloxone significantly blocked the cognitive-mediated analgesia induced by specific contexts and strong or weak thermal stimulation (Fig. 3), implying that the context-based analgesic effect in rats mainly relies on expectation of a strong or weak stimulation based on previous experience rather than conditioning.

Context-based analgesia, or placebo analgesia, is of great interest for future clinical applications. Not only is qualitative research on humans needed, but mechanistic research in animals will also help. This novel rat model will shed new light on our understanding of the brain mechanisms underlying cognition-modulated analgesia as well as non-drug-induced, expectation-dependent placebo analgesia, which will help develop improved treatment strategies for patients in pain.

In conclusion, we have established a non-drug dependent, context-based analgesia model in rats that is endogenous opioid-dependent. The PFC, particularly the PL and the IL cortices, is involved in this analgesic effect. Our study provides a new model for studies of the cognitive modulation of pain as well as placebo-like analgesia in animals.
